# Pain
*Based on an address to the Bristol Medico—Chirurgical Society.


**Published:** 1960-10

**Authors:** Leslie Hill

**Affiliations:** Consultant Physician, Bath Clinical Area


### PAIN*

BY

LESLIE HILL, M.D., F.R.C.P.

*Consultant Physician, Bath Clinical Area*

Pain is perhaps the commonest symptom for which our help is required. Its interpretation, assessment, and alleviation is the main problem facing the doctor. The word is used for two separate, if often related, phenomena. The first is the unpleasant sensory experience that results from bodily injury or disease. The second is an emotional disturbance which usually accompanies and colours the sensory experience of pain, but which may on occasion result from purely psychological disorders.

### THE ANATOMY AND PHYSIOLOGY OF PAIN

(*a*) *Pain pathways.* Pain fibres from the trunk and limbs enter the spinal cord in the posterior root, ascend on the same side, usually for about three segments, and end in the posterior horn cells of the same side. The next relay crosses immediately to the lateral spinothalamic tract of the opposite side and ascends in this tract through the cord and medulla to the pons, where it joins the lateral portion of the median fillet, passing upwards to the ventral nucleus of the thalamus. The next relay passes through the posterior limb of the internal capsule and the corona radiata to end in the post-central region of the cortex. Pain fibres from the face enter the pons through the Vth cranial nerve and turn downwards in the descending tract of the trigeminal nerve to end in the nucleus of this tract, which descends as far as the second cervical segment of the cord. The next relay crosses in the medulla and ascends in the quinto-thalamic tract to join the spinothalamic tract, and so reaches the ventral thalamic nucleus.

(*b*) *Cortical projection of pain.* Impulses travel from the ventral nucleus of the thalamus to the cortex. From experiments on monkeys, it seems that a definite topical plan exists, whereby fibres from the inner portion of the nucleus end in the lower part of the gyrus, those from the outer portion in the paracentral region, and those from the middle portion in the intermediate part of the post-central convolution. This does not, however, tell us how pain is appreciated. It is only possible to gain insight into this by assigning functions to structures in the light of the defects produced when a portion of the pain system is destroyed. Appreciation of pain arises from structures at or above the mesencephalon; a lesion of the upper pons will abolish peripheral pain. Small lesions of the cortex produce little or no impariment of appreciation of pain. Larger lesions, however, will at times produce hypalgesia of mild or severe degree. More frequently they produce an inability to distinguish between pains of different qualities. In a case of hemispherectomy cutaneous sensation was lost everywhere except in the face; joint sense was not affected. It must, therefore, be concluded that extensive cortical lesions may produce varying degrees of hypalgesia, with the face much less affected than the rest of the body.

(*c*) *The mechanism of common sensibility.* Weddell and his colleagues have recently drawn attention to the impossibility of equating the four commonly recognized modalities of cutaneous sensation—touch, warmth, cold, and pain—with four separate and distinct kinds of nerve endings in the skin. To determine whether a particular nerve

*Based on an address to the Bristol Medico—Chirurgical Society.

terminal is related to a particular sensation such as pain may appear a straightforward question, but it is not. There are two theories. The commonly held view is that pain is subserved by a specific set of pheriperal sensory nerves and their specific nerve-endings. More recent work has cast doubt on the presence, certainly in hairy skin which covers the greater part of the body, of circumscribed nerve endings such as Meissner's corpuscles, Krause end-bulbs, and so on. Instead, there are widespread arborisations of fine, freely-ending filaments which interweave, the so-called free nerve endings. Lele's work suggests that sensation of heat is in some way connected with the pattern of the discharge evoked from the skin, and not just to the discharge from specific nerve endings. It has also been shown that the mode of innervation of hair on the rabbit's ear is multiple and complex, each hair being served by at least two dorsal root axons. The average is about six, and there may be as many as twenty involved. It has been calculated that every root axon could be made to discharge simultaneously if no more than one quarter of the total hairs on the area were stimulated. This can only mean that hair messages are coded for transmission, and reach the central nervous system as sets of spatially and temporally dispersed action potentials which are then presumably decoded. There can be no question of each hair having a private and exclusive line.

Weddell brings further evidence and argument to suggest that free nerve-endings and the nerves subserving them in the skin are arranged in a similar way. His work has shown that all the modalities of senation are independent of their so-called specific nerve-endings, Meissner corpuscles and the like, and suggests that pain, for example, is a mathematical summation of the temporal and spatial distribution of impulses passing centrally along peripheral nerves.

(*d*) *The pain of inflammation.* There are probably various factors concerned, which include increased tension from rapid distension of soft tissues, increased accumulation of metabolites (the raised local temperature favouring their production), and occlusion of the circulation which may act by hindering the removal of metabolites or by producing anoxia of the tissue.

Keele's work with fluid drawn from blisters, pleural effusions, or rheumatoid joints, which causes pain when applied to a raw surface, suggests that tissue damage brings about the release of an active proteolytic enzyme, which in turn acts upon the plasma to form pain-producing substance (P.P.S.). Thus in addition to the effects of tension, metabolites, and ischaemia, pain in inflammation may be caused by the release of 5-hydroxy-tryptamine, histamine, and P.P.S.

(*e*) *Vascular causes.* It is not disputed that ischaemic pain is due to anoxia, whether that be relative, in the presence of excessive muscular activity (intermittent claudication), or absolute from vascular spasm (angina pectoris). It is not yet established whether oxygen deficiency either leads to the accumulation of metabolites or interference with their removal. In reference to visceral pain, it is of interest that William Harvey performed several experiments on the son of Count Montgomery, a friend of Charles I. The child had suffered a severe accident, which left him with his heart exposed, and its beating could be easily seen and felt. Touching the heart produced no sensation. It was therefore assumed that what had been regarded since Aristotle as the "seat of the soul" was insensitive. What then was the cause of cardiac pain? We now know that it is ischaemia.

(*f*) *Multiple causes.* The pain of hiatus hernia is usually felt below the sternum and may radiate to the back. Sometimes, however, it occurs higher up and is identical with angina; in which case it has been noted that successful repair of the hernia appears to cure the pain, though it may recur in the same form several years later. There is experimental evidence for the view that this pain originates in the heart, and is induced by a disordered oesophagus. The introduction of an inflated balloon into the lower end of the oesophagus of healthy young anaesthetised dogs causes a reduction of coronary flow of up to 50 per cent. It is thus clear that if the coronary flow is already reduced by atheromatous changes, further reflex reduction would be likely to cause angina. The fact that hiatus hernia in young persons never causes typical anginal pain with radiation to the arm would seem to support this. A similar association of anginal pain with gall-bladder disease exists clinically. Again, experimental mechanical stimulation of the bile ducts outside the liver has been shown to reduce coronary flow by at least 50 per cent. Improvement in the angina which follows removal of a diseased gall-bladder is often dramatic. In the same way, therefore, gall-bladder disease is more likely to cause angina when there is a pre-existing reduction in coronary flow from atheroma.

### CLINICAL ASPECTS OF PAIN

In these days of high-powered diagnostic instruments the importance of a careful analysis of a history of pain is insufficiently stressed; the process of unravelling an often confused description is all too time-consuming to be popular in a busy out-patient clinic; but it is invaluable when pain is the sole complaint. The experienced clinician automatically analyses the quality of pain by following a definite thought-plan: "Pain—new or chronic? Patient—healthy or ill?" He observes and subconsciously records the walk of the arthritic, the bland expansiveness of the unoccupied widow, the sweat on the brow of the anginal traveller, the paunch of the diabetic with neuropathy, the hang-dog, truculent attitude of the compensation-seeker. He has taken all this into account before he considers the details of site, distribution, type, and depth of pain. He remembers that each man's conception of pain is made up of his own personal experience, together with such accounts of their pain as other people can give him. As Kellgren points out, even the trained investigator acting as his own guinea-pig finds it difficult to translate sensory experience into words. How much more difficult for the average out-patient, especially when the emotional component is high! And the appreciation of pain cannot be separated from the emotional make-up of the individual. Stick a pin into one man and he says stolidly "It's a prick"; another will scream and withdraw violently. The indifference of the leucotomised patient to fully-perceived pain can in a homely way be exemplified by comparing one's own reaction to a painful injury after a champagne and brandy-lightened dinner with that to a similar injury after a cup of hot black coffee on a cold winter morning.

### *Pain in children*

In the neonatal period, we believe there is a relative indifference to pain. The baby can be circumcised without an anaesthetic, provided it is happily sucking a bottle. Later on, after a month or two of life, pain is felt, but imperfectly localized. Thus pains from abdominal colic, otitis media, or bone inflammation may all produce the same reaction—screaming, drawing up of the legs, vomiting and convulsions. The drawing up of the legs may be translated by the mother as "the wind"; beware of accepting her diagnosis!

Gradually, through childhood, localization becomes more distinct, though still imperfect, especially in respect of the abdomen. At this age inability to localize pain in the head is not a sign that it is psychogenic. Suspect, rather, the child who complains of "dreadful pain". Again, a child with a sore throat rarely complains of it; he goes off his food and may vomit. This is not necessarily a disadvantage; you will remember the great Dr. S. J. Gee's advice to young doctors—"Eyes first and much, ears second and little, hands not at all". In early infancy, then, do not expect pain or its accompaniments. Later on, do not expect accurate localization. Let your examination be thorough.

In appendicitis in childhood pain is always present, but the difficulty is that so many other conditions may cause pain in the belly. If our examination is cursory, and if we are not prepared constantly to be on the watch for this possibility, the child may slip away; and the younger the patient the more rapid is that process. Pericarditis is now a rarity in childhood, but no one having seen it will forget the desperately ill, toxic child whose illness throughout has been unaccompanied by complaints of pain.

The general reaction of children to pain is good. Like dogs, they tend to crawl away and get better. This tendency, of course, is modified by the mother's reaction to the situation, and by the association in their minds of doctors and pains, and often by fear of what the doctor may do. How often we are greeted by "You are not going to look at my throat, are you?"

Apley has pointed out that there are suggestive links between the usual, recurrent, non-organic pains of childhood, whether they occur in the limbs, abdomen, or head (the only common sites in this age group). In studies carried out over a number of years interesting similarities were observed, not only between children who made similar complaints, but also between their families. He suggests that those pains may be grouped together as expressions of a reaction-pattern, which is usually associated with emotional disturbances both in the child and in other members or the family.

*Pain in the aged*

On the whole the aged show less outward expression of pain, less tendency to be restless or to cry aloud, unless they be demented when the pain threshold is raised. Some of the pains they suffer are devastating—the particular hell of post-herpetic neuralgia; another hell for *tic douloureux;* and a third for ischaemic pains in the limbs with "the jumps". But perhaps the "horizon pain" is worst of all—the bleak, long, comfortless night across the ocean of sunless misery.

On the other hand, the old ones tend to complain less of their peptic ulcers, and hardly at all of toothache even in the presence of hideously infected teeth. Again, the older the patient the more likely it is that circumstances will permit him to rest the part; in fact pain may be a cause of immobility in old age.

In addition to post-herpetic neuralgia and *tic douloureux*, it is well to be on the look-out for giant-celled arteritis (including temporal and cranial arteritis, which may of course present typically with temporal or occipital headache) which may begin insidiously with pyrexia, malaise, and "fibrositic" pain in head, neck, or shoulders. It is important to recognize this disorder because of the danger of sudden blindness. Osteoarthritis of the hip is another geriatric pain problem; if pain is present at rest or in the night, radiotherapy will sometimes help.

*The Rheumatic Group*

Pain is *the* dominant complaint of all rheumatic sufferers. In some form or other it is constantly forcing itself to the surface of any activity, and when it abates its room is as conspicuous as its presence. It may for long be the only symptom, recurring for varying periods without any other evidence of disease, and without being in itself dangerous or seeming to provide a protective mechanism. Its interpretation, assessment, and alleviation is the main problem facing the doctor.

(*a*) *Neuralgia*. So-called characteristic features include the paroxysmal nature of the pain, absence of clinical signs, and absence of pathological findings. None of these stands up to critical assessment. *Trigeminal neuralgia*, the prototype, the neuralgia *par excellence*, pure pain, has already been alluded to. Those who favour a strict differentiation between neuritis as an organic disease and neuralgia as a functional disease draw their main support from this. How does it fit into the criteria mentioned above? Not at all. It is not always paroxysmal in the mild cases; minor sensory changes may be present in the direction of reduction in sensation; modern methods of staining do show changes in the cells of the Gasserian ganglion; trigger zones are not always present; and it may be associated with herpes simplex. The existence of a *true occipital*

*neuralgia* is also doubtful, for the pain is either a neuritis from root pressure in cervical spondylitis or a myalgia of vascular origin. In both cases rest, warmth, and a firm pad in the nape of the neck at night are more rewarding in my experience than resort to the physiotherapy department or the chiropractor. A simple rubber collar is effective for the same reason. Remember that manipulation of the neck can precipitate vertebral thrombosis.

(*b*) *Acroparaesthesia and the carpal tunnel syndrome.* One of the commonest types of patient seen in the rheumatic (and, I suspect, the neurological) clinic is a tired worn-looking woman of 40–60 years, who wakes up in the night with painful paraesthesiae in the fingers, and pain in the forearm, sometimes extending as high as the shoulder. The fingers feel stiff and swollen, and are clumsy. There are normally no physical signs, except very occasionally impaired sensation over the tip of one or two digits. The term "acroparaesthesia" was introduced by Schultze in 1893 to describe these largely nocturnal symptoms in women. In 1945 Walshe put the problem on a sounder basis, suggesting a mechanical cause—traction or compression of the brachial plexus or the subclavian artery, or both, between the clavicle and the first rib, as a result of sagging of the shoulders from fatigue. Obliteration of the radial pulse by dragging down the arm is usually easily obtained. Middle-aged women, often of the middle class, are the chief sufferers. Is it because they have to tackle housework to which they are not accustomed and cordially dislike?

A special variety is seen in the last trimester of pregnancy, usually after the 32nd week; it clears up within hours of delivery, and can be relieved earlier by promoting water loss. Some of these women relapse later on, probably for two reasons—increase in weight, and the extra activity required in looking after the baby. The essential principle of treatment is always a preliminary period of rest in bed. Later, when rested, help may be obtained from heat (to promote muscle relaxation), light massage, and bracing exercises for the neck and shoulders. In the early stages all forms of physiotherapy aggravate the pain.

The recognition of the carpal tunnel syndrome as a cause for some of these symptoms constitutes a great advance in the elucidation of pain in the upper limb, and has to some extent shifted the onus from the costo-clavicular space.

My own view is that sensory or motor disturbances resulting from pressure in the costo-clavicular space are precise in pattern, corresponding to the distribution of the 1st dorsal nerve. In severe cases there may be a certain amount of over-spill, but the symptoms are rarely vague or widespread. When the pain is described as affecting the arm and the forearm, perhaps spreading into the neck or the side of the chest, without muscle wasting, suspect spondylitis, with or without postural faults. When the area affected is confined to the forearm, and especially to the outer border of the hand, suspect pressure on the medium nerve in the carpal tunnel. In the latter case, a careful examination will reveal slight wasting on the outer side of the thenar eminence with loss of sensation on the palmar aspect of the index and middle fingers. Unfortunately, cervical spondylosis and sagging of the shoulders are extremely common in women of this age, and it is not long before a psychogenic factor becomes superimposed.

(*c*) *Backache.* Space does not permit a proper consideration of shoulder-girdle pain or of sciatica, but I must spare a few lines to marshal some arguments against the absolute supremacy of the inter-vertebral disc in the doctrine of backache and sciatica.

(i) Protusion of the intervertebral disc in the lumbar region is very common in later life and is a frequent autopsy finding, without any history of pain. We may have pain without a "disc", and a "disc" without pain.

(ii) The evidence is against the importance of trauma. Patients in general, not excepting those with sciatica, have an overwhelming desire to attribute their troubles to external factors, particularly trauma. Often, on close questioning, the pain is found to have occurred before the accident.

(iii) In many cases at operation (26 out of 100 in Wartenberg's series), no disc protrusion has been found; and it is well to remember that in simple neuritis laminectomy helps by virtue of its decompressive effect.

(iv) Some patients complain that their sciatica followed exposure to cold.

(v) Sciatica occurs with herpes zoster, or following acute infective states such as brucellosis or tonsillitis.

(vi) Neuro- and orthopaedic surgeons tend to base their views on severe and intractable cases which have resisted all other forms of treatment. They do not see the mild cases, which may be transient. Three out of four cases recover quickly with medical treatment (warmth, rest, analgesics, and a firm bed). It is unlikely that these remissions depend upon the retraction of a prolapsed disc. Sciatica is, in my experience, a benign affair, and clears up within two or three weeks. It is to be fervently hoped that this unfortunate intermezzo of disc hysteria will now abate. As the French say "On affaiblit toujours tout ce qu'on exagère". Two other quotations spring to mind: "We were better off with lumbago and sciatica when we knew nothing about discs" (Brailsford); and "Surgery of the intervertebral disc is major surgery, and the penalty of error is severe" (W. T. Mixter).

(*d*) *Panniculitis*, or "tension syndrome" is thought to be due to distension with fluid of the fatty tissues where they lie within an indistensible fibrous covering. The main factor is vascular, but there are probably endocrine influences. The patient is often obese, and the areas affected include the outer parts of the shoulders and thighs and the internal fat pads of the knees. The syndrome, if it occurs before the menopause, is generally most painful in the pre-menstrual periods. A characteristic sign is the peculiar *peau d'orange* effect when the thickened skin in the painful area is picked up. The prognosis is good if adequate treatment is given early and persevered with; this includes a reducing diet, salt restriction, exercises, hydrotherapy, and massage.

(*e*) *Fibrositic pain.* I should like to suggest that after the separation of such definable entities as tendinitis, bursitis, panniculitis, fat herniation, and psychogenic rheumatism, there remains a group of individuals with a diffuse or localised soft tissue pain and stiffness in whom, in the absence of clinical, laboratory and X-ray abnormalities, the diagnosis of "fibrositis" seems justifiable. The earlier concept of local inflammatory change in muscle and connective tissue is now quite discredited, and should be discarded. I should like to suggest boldly that the fundamental factor is vascular, and that the pain in muscles and soft tissues is ischaemic pain. Interference with blood supply may result from spasm or from atheromatous change in the arterioles, or from a combination of the two. It is a matter of observation that many chronic rheumatic patients are elderly and suffer from other results of circulatory disturbance. Equally, many show the stigmata of tension, anxiety or depression. Vascular spasm is probably reflex, and can be brought about by a wide variety of stimuli, which include postural faults, muscle over-fatigue, temperature and humidity variations, nerve root pressures, and (commonest of all) psychogenic influences. Elliot, in 1952, showed by electromyography that the tender areas found in muscles are due to localized spasm. The tender spots, or so-called "fibrositic nodules" are usually, but not always, of this type. Nevertheless, the diagnosis of fibrositis is beset with pitfalls. Here are a few: *With fever*, think of rheumatic fever (the forgotten disease), subacute bacterial endocarditis (the missed disease), brucellosis and glandular fever (the over-diagnosed diseases). *With wasting*, think of cancer and any of the collagen disorders (the fashionable diseases), temporal arteritis and hypernephroma (the undiagnosed disease). *With anaemia*, think of these and of chronic renal failure. Do not forget the pains of metabolic bone disease.

The connection between steatorrhoea and osteomalacia is well known, but the steatorrhoea may for long be occult and the patient may complain only of generalised fibrositis-like aches and pains, with tenderness of the bones and a waddling gait.

*Psychogenic Pain*

Is the pain real? To the patient all pain is real, except for the true malingerer. If we identify the word "real" with adequate organic cause, then there are pains which are definitely not real. And here I would remind you that it is indefensible to call any pain or any symptom "neurotic" simply because you cannot immediately find an organic explanation, or, which is even more pertinent, because there are surrounding circumstances tending to neurosis. There must be both absence of organic signs and the presence of other stigmata of anxiety or hysteria. Psychogenic causes, in my experience, predominate in the non-articular group of rheumatic diseases, properly designated "rheumatics". How shall we hope to recognize psychogenic pain? The patient projects his unhappiness into some part of the body where he keeps it quite cheerfully as an "agonizing pain". These pains are often bizarre in distribution and described with drama snd detail. The more ubiquitous the pain, the more is it likely to be neurotic. Conversely, when a patient month in and month out points to one place you can be reasonably certain there is an organic cause. There are the people (the anxiety neurotics) in whom mental tension expresses itself in widespread muscle spasm —they move awkwardly and stiffly, contracting agonists and antagonists simultaneously; this leads to fatigue and painful muscle strain. In hysteria, which is rare, the pain is used to achieve a particular personal aim, usually that of escaping from a difficult situation or fulfilling a desire. In studying the patient, other psychogenic disturbances or obvious constitutional inadequacy will appear. Then, there is usually a history of multiple consultations, innumerable investigations, months of physiotherapy and repeated operations—and, in spite of this, or because of it, the patient will often complain of the unsuccessful treatment carried out by former doctors, and of their negligence and ignorance. Remember that your turn, too, will come. You will be lucky if you do not end up by introducing yet another iatrogenic symptom. We must also remember that pain may become an "old friend", and there are occasions when we do the patient a disservice by attempting to undo his solution of an agonizing problem.

We must not be afraid to tell the patient, after a careful examination, our findings and conclusions. I have been astonished to note how often, if the explanation is given quietly and in a kindly, sympathetic manner, the psychogenic patient is willing to accept it, and this understanding of the problem of tension and spasm as a cause for pain is more than half the battle. Beware of the alternative line of sending such patients to swell the ranks of chronics littering the physiotherapy departments all over the country. In these departments it has long been recognized that spastic areas in muscles are the site of pain and that such pain can often be temporarily relieved by the application of heat and massage, or by the injection of a local anaesthetic; but only temporarily, for it requires much more than this to get to the root cause and break the vicious circle. This is not to suggest that physiotherapy has no part to play in the management of the psychogenic rheumatic; provided that the patient fully appreciates that it is only part of the plan of treatment and provided also that the importance of achieving relaxation is fully understood, it can be useful.

*Pain—Friend or Foe?*

I should like now to strike a philosophical note and introduce a conception of pain as a physiological function in a pathological environment. That pain, long continued, wears the sufferer down, none can deny. Yet pain alone could be harmless. It is the secondary effects that do the harm—shock from sudden pain, inactivity, depression, and fear of prolonged pain. Of all pains, none is more terrible than that of invasive cancer. By contrast, consider the pain of trigeminal neuralgia. Whereas the one keeps up its relentless barrage right up to the moment of death, the other, exquisite agony though it may be, will suddenly lift, and the victim, seconds earlier driven almost out of his wits, becomes at once his normal self. May I be privileged to remind you of the contribution of a distinguished West Country man, himself both physician and philosopher, to the literature of pain. John Locke was born in 1632 at Wrington, at the foot of the Mendips. His vivid description of a paroxysm of trigeminal pain in the Countess of Northumberland, wife of Charles's trusted ambassador in Paris, is a veritable masterpiece, and I cannot imagine that it has ever been equalled for its objective and scientific clarity, both relieved and emphasized by the warmth of Locke's understanding. In describing the incident to his friend, Dr. Mapletoft, he was giving the first account in Europe of *tic douloureux*. Here, surely, is pure pain, unalloyed by the consequence of organic disease. It has the simplicity of physical signs in children, uncomplicated as they are by the legacies of earlier pathology.

I believe that Philip Hench's most important contribution to rheumatology was his concept of the reversibility of rheumatoid arthritis (as exemplified in jaundice and pregnancy). I suggest that this concept be extended to pain and that we think of its reversibility and, in a sense, its essential benignity. This latter is not a quality usually attributed to pain, but it might be granted to a function that is strictly physiological. Where there is pain, there is also life. Death and the grave are painless. We see a charade of this in rheumatism; when the process of destruction has gone beyond a certain point pain disappears, as in advanced stages of arthritis mutilans. So long as the injured tissues can respond physiologically to disease by giving rise to pain, then reversibility and recovery are still possible. Pain is a sign of life. A similar analogy comes to mind when we contrast dental pain and its subsidence with death of the tooth.

We can sometimes envisage pain as a local protective mechanism, interference with which may encourage a damaging level of performance and bring about permanent destructive changes. As examples of this, I well remember two patients with recurrent effort angina in whom pain, continuing in spite of all the usual measures, was finally controlled by stellate ganglion block. Within a short time both patients were in serious heart failure, probably as a result of the extra exertion hitherto prevented by the pain. Steroids in the treatment of rheumatoid arthritis, by relieving pain and spasm, often permit a level of activity for which the joints are not prepared or the muscle tone adequate, with the result that destruction proceeds apace. Recently, Evans and his colleagues have drawn attention to the danger of intra-articular hydrocortisone which, by its anti-phlogistic and analgesic effect—often quite spectacular—permits a damaging level of activity so that destructive changes occur, which, if not obvious clinically, are only too obvious in serial X-rays.

### TREATMENT

(*a*) *Analgesics*. I agree with McIlwain that "opium has probably contributed more than any other preparation to faith in the healing art and belief in the feasibility of lessening human ills by administered drugs". Knowledge of analgesic drugs has been limited by lack of any reliable method of assessing their effects. Recent work by Wolff, Hardy, and Goodell has greatly added to our knowledge, They devised methods of quantitively assessing the action of analgesics and analysing the various factors that modify their effectiveness. All the commonly used analgesics (morphine, codeine, alcohol, aspirin) were found to raise the pain threshold in that order of effectiveness, and for each there was a dose producing a maximum or ceiling effect, which was not exceeded by further increasing the dosage. In the course of their studies, they became aware of other valuable effects, beyond the mere heightening of pain threshold, effects particularly upon the mental state of the individual. It seems clear that, in the absence of anxiety regarding its source, pain is comparatively well tolerated.

The effect of the mental state upon the pain threshold is well recognized by drug manufacturers and, of course, is the main reason for the relative effectiveness of morphia. Pain threshold can be raised by as much as 30 per cent by suggestion through placebos. Greater rises, but lasting for a shorter time, have been obtained by distraction. These considerations call for a change of emphasis in regard to the mechanism of action of pain-killing drugs, and may perhaps explain in some way the popularity of various faith-healing cults. Analgesic drugs are useful in the treatment of pain, but their full effect can only be achieved when the physician commands the full confidence of his patient.

(*b*) *Surgery*. Intractable visceral pain is often due to the direct extension of the morbid process into the spinal nerves. In such cases, section of the posterior spinal nerve roots (posterior rhizotomy) may be a valuable procedure, provided that the area affected is limited to a small number of spinal segments. When a large number of spinal roots are involved cordotomy may rarely be justifiable. Posterior rhizotomy is a serious and hazardous operation. It has its greatest use in the treatment of severe localized pain in the neck and trunk, for example in bronchogenic carcinoma beginning in the apex of the lung, carcinoma of the breast invading the chest wall, and intractable angina.

Surgical relief of *tic douloureux* should, if possible, be conservative. Section of the sensory root behind the ganglion under the best of conditions leaves an uncomfortable, stiff, swollen feeling in the face and tongue. 5 per cent may get facial paralysis; 5 per cent a persistent burning sensation in the anaesthetic area. The patient's gratitude to the surgeon does not last. A better method is the use of alcohol injections into the appropriate branch of the trigeminal nerve.

In the past leucotomy has been advised for pain only when all other possible methods of treatment have failed. This as, McKissoch has said, is bad practice, and few will disagree with his view that what in a final analysis proves the most beneficial operation should be the initial one. Elithorn, Glithero, and Slater have carefully studied the results of this operation in twenty-five patients with intractable pain followed up for from two to six years. They confirm the previously held view that leucotomy does not affect the perception of pain and their findings suggest that the immediate autonomic and behaviour disturbances aroused by a new painful stimulus are, if anything, greater after leucotomy damage. On the other hand, such disturbances are less prolonged and less readily aroused by fear of a repetition of the pain. It must be remembered that leucotomy, while causing a shock effect similar to that produced by electroconvulsive therapy, with the breaking up of established moods particularly of depressive type, also produce a focal and persisting neurological deficit resulting in changes in the disposition of the patient, in particular a reduction in the tendency to become anxious. Typically, in such cases the patient still complains of pain, but shows at the same time an inappropriate cheerfulness. Ordinarily, this would suggest hysteria, and, in fact, in Elithorn's series, those patients showing hysterical features before operation became more hysterical afterwards.

In selecting cases for this operation, the main indication is pain in association with an excessive psychological reaction in the anxious, introspective, depressive type. The more definite the physical pain, then the less likely is it that leucotomy will help. Since the risk of either excessive personality-change, post-operative epilepsy, or operative death amount to about 10 per cent, it is clear that damaging operations on the frontal lobes provide no satisfactory answer and are only justified if a direct attack upon the pain is impossible.

